# Predicting identity dissociation using childhood maltreatment and genetic variation in the stress-response gene *FKBP5*: a machine learning analysis

**DOI:** 10.1038/s41598-026-42512-0

**Published:** 2026-03-06

**Authors:** Leonhard Kratzer, Hans Knoblauch, Abigail Powers, Seyma Katrinli, Vasiliki Michopoulos, Negar Fani, Charles F. Gillespie, Tanja Jovanovic, Kerry J. Ressler, Alicia K. Smith, Bertram Müller-Myhsok, Stefan Tschöke

**Affiliations:** 1 Department of Psychotraumatology, Clinic St. Irmingard, Prien am Chiemsee, Germany; 2 Clinic for Psychiatry and Psychotherapy I (Weissenau), Ulm University, Ulm, Germany; 3 Centre for Psychiatry Suedwuerttemberg, Ravensburg, Germany; 4 Department of Psychiatry and Behavioral Sciences, Emory University School of Medicine, Atlanta, GA USA; 5 Department of Gynecology and Obstetrics, Emory University School of Medicine, Atlanta, GA USA; 6 Department of Psychiatry and Behavioral Neurosciences, Wayne State University School of Medicine, Detroit, MI USA; 7 Department of Psychiatry, McLean Hospital, Harvard Medical School, Belmont, MA USA; 8 Research Group Statistical Genetics, Max Planck Institute of Psychiatry, Munich, Germany

**Keywords:** Identity dissociation, Childhood maltreatment, Gene-environment interaction, *FKBP5* haplotypes, Biomarkers, Diseases, Genetics, Medical research

## Abstract

Identity dissociation is challenging to detect and treat, and its etiology remains incompletely understood. Childhood maltreatment and FKBP5 polymorphisms, which modulate the stress response, may contribute by disrupting the integration of autobiographical experiences essential for identity development. We examined whether gene-environment interactions involving childhood maltreatment and *FKBP5* polymorphisms predict clinically significant identity dissociation. In a cohort of *N* = 377 participants, we assessed childhood maltreatment and identity dissociation using validated questionnaires and genotyped CATT haplotypes within *FKBP5* linked to stress reactivity. Identity dissociation was dichotomized using an established clinical threshold. An elastic net regularized logistic regression model incorporating maltreatment subtypes, CATT haplotype count, and their interactions was trained (*N* = 194) and validated (*N* = 183). Model performance was evaluated using the area under the receiver operating characteristic curve (AUC), sensitivity, specificity, and Matthews correlation coefficient. Decision curve analysis assessed clinical utility across varying risk thresholds. The model demonstrated fair discrimination (AUC = 0.709) with 58.6% sensitivity and 79.9% specificity. While the positive predictive value was modest (35.0%) due to the low prevalence of identity dissociation (15.9%), decision curve analysis revealed a net clinical benefit across a broad range of threshold probabilities (6–76%), indicating practical utility for risk stratification in clinical settings. The negative predictive value was 0.91. These findings provide initial evidence that gene-environment interactions between childhood maltreatment and *FKBP5* variation may contribute to the risk of identity dissociation. While predictive precision remains limited, this study demonstrates the feasibility of applying machine learning approaches to dissociation and highlights the need for further research into their traumatic and biological underpinnings to improve detection, prevention, and treatment.

**Supplementary Information:**

The online version contains supplementary material available at 10.1038/s41598-026-42512-0.

## Introduction

Within the spectrum of dissociative phenomena, little is known about the genetic contributions to specific severe forms such as identity dissociation, defined as disruptions or discontinuities in the perception of oneself as a consistent person over time^1,2^. Identity dissociation characterizes dissociative identity disorder, which has been repeatedly linked to childhood trauma^3^, as well as to reduced narrative identity coherence, metacognitive alterations, and memory disturbances^4–6^. Recently, we identified a relationship between childhood traumatic stress, the CATT haplotype (a variant within the *FKBP5* haplotype cluster), and identity dissociation^7^.

Building on this association, we hypothesized that the interaction of childhood maltreatment with the CATT haplotype, a genetic variant associated with an overregulated stress response via the hypothalamic-pituitary-adrenal (HPA) axis, can *predict* the development of clinically significant identity dissociation. To test this, we employed a machine learning framework to evaluate the predictive power and clinical utility of this gene-environment interaction. This shifts the focus from a simple association to a clinically actionable predictive model, representing a critical advance over our previous work and the broader literature.

## Methods

### Ethics

All study procedures were approved by the Institutional Review Boards of Emory University School of Medicine and Grady Memorial Hospital.

This study was performed in accordance with the ethical principles outlined in the Declaration of Helsinki and its later amendments^49^. Data handling and processing conformed to all applicable Health Insurance Portability and Accountability Act rules. Data was de-identified to preserve both anonymity and confidentiality. Furthermore, this study was conducted under the research exception provisions of the Privacy Rule, 45 CFR 164.514(e), and was therefore exempt from institutional review board informed consent requirements. No new human participants which had not been previously included in prior studies were recruited for this study.

### Data availability

The datasets used and/or analysed during the current study are available from Abigail Powers Lott (Co-Director, Grady Trauma Project) on reasonable request.

### Study participants

The study sample comprised *N* = 377 participants drawn from a large-scale study on the impact of trauma-related risk factors for post-traumatic stress disorder and related comorbidities in a high risk, trauma-exposed urban population of Black Americans in Atlanta, USA, with a sample size of *N* = 11,524^8,9^. Inclusion criteria for the current study were the experience of at least one traumatic event, assessments of both childhood maltreatment using the childhood trauma questionnaire (CTQ)^10^ and dissociation using the multiscale dissociation inventory (MDI)^11^, as well as genetic data on the presence of the CATT haplotype. The only exclusion criterion in the overall study was psychiatric hospitalization within 30 days of enrollment. Participants were recruited between 2011 and 2016 at Grady Memorial Hospital, Atlanta, Georgia, USA.

The sample consisted of 334 Black women (88.6%) and 43 Black men (11.4%) with a mean age of 40.9 years (*SD* = 11.8). Demographic characteristics such as education, relationship status and income can be found in Table 1.

**Table 1:** Demographic characteristics of all study participants.

	*N*	%
Relationship status		
Single, never married	216	57.3
Married	37	9.8
Divorced	80	21.2
Separated	19	5.0
Widowed	7	1.9
Domestic partner	18	4.8
Education		
Less than twelfth grade	80	21.2
12th Grade/high school graduate	111	29.4
General educational development	16	4.3
Some college or technical school	95	25.2
Technical school graduate	28	7.4
College graduate	38	10.1
Graduate school	9	2.4
Employment		
No	264	70.2
Yes	112	29.8
Disability		
No	305	81.1
Yes	71	18.9
Arrests		
No	182	48.3
Yes	195	51.7
Approximate household monthly income		
$0–249	60	15.9
$250–499	26	6.9
$500–999	102	27.1
$1000–1999	105	27.9
$2000 or more	78	20.7

### Procedure and materials

Dissociative symptoms were assessed using the MDI^11^, a comprehensive self-report measure that captures various dimensions of dissociation, including emotional constriction, disengagement, depersonalization, derealization, memory disturbance, and identity dissociation. The subscale identity dissociation uses items asking for experiences like “Having different people inside of you with different names”, “Feeling like there was more than one person inside of you”, and “Switching back and forth between different personalities”. The MDI comprises 30 items, each rated on a 5-point Likert scale ranging from 1 (“never”) to 5 (“very often”). Subscale scores are transformed into *T*-scores based on normative data which allows for the evaluation of symptom severities across the respective dissociative domains. For the present study, a *T*-score > 70 on the identity dissociation subscale according to the cut-offs reported in the manual was used as the threshold for clinically significant identity dissociation.

Childhood maltreatment exposure was assessed using the CTQ^10^. This 28-item self-report instrument evaluates five types of childhood maltreatment: emotional, sexual, and physical abuse, as well as emotional and physical neglect. Items are rated on a 5-point Likert scale, ranging from 1 (“never true”) to 5 (“very often true”). Subscale scores were calculated and used as continuous predictors of clinical outcomes for this study. Participants completed the MDI and CTQ assessments during research visits with study staff.

### Data analysis

All analyses were conducted using R (version 4.5.0^12^)^53^ with the packages *glmnet*^13^, *mltools*^14^, *rmda*^15^, *ggplot2*^16^, *pROC*^17^, and *apaTables*^18^. A binary classification model using elastic net regularized logistic regression was developed to predict the presence of clinically significant identity dissociation based on childhood maltreatment and genetic data. The outcome variable was identity dissociation, dichotomized using a *T*-score threshold of > 70 (0 = absent, 1 = present). Predictors included five types of childhood maltreatment (emotional neglect, physical neglect, emotional abuse, physical abuse, sexual abuse), biological sex, the number of CATT haplotypes, and five interaction terms between each maltreatment type and the number of CATT haplotypes. To ensure model transparency and address the “black box” concern often associated with machine learning, we extracted both raw and standardized coefficients. Standardized coefficients were calculated to allow for a direct comparison of the relative contribution of each maltreatment subtype and its interaction with the *FKBP5* genotype to the prediction of identity dissociation. Model training was performed using a previously analyzed dataset (*N* = 194)^7^.

Nested cross-validation was employed to tune the α parameter of the elastic net penalty, testing values from 0 (ridge regression) to 1 (lasso regression) in increments of 0.1. For each α, 5-fold cross-validation was performed using the area under the receiver operating characteristic curve (AUC) as the performance metric. The α yielding the highest mean AUC was selected for final model training. The corresponding optimal λ (regularization strength) was identified using 5-fold cross-validation, also using AUC as the criterion.

The model’s predictive performance was evaluated using an independent validation dataset (*N* = 183). We computed the AUC, optimal threshold (based on Youden’s Index), classification accuracy, sensitivity, and specificity. Additionally, the Matthews Correlation Coefficient (MCC) was calculated, as it offers superior performance over Cohen’s κ for evaluating binary classifications^19,20^.

To assess clinical utility, decision curve analysis (DCA) was conducted^21^. DCA quantifies the net benefit of using the prediction model across a range of threshold probabilities by comparing its performance against default strategies of treating all or no individuals as positive cases, thereby evaluating whether the model offers meaningful advantages for guiding clinical decision-making.

For further analyses, we calculated a binary childhood maltreatment exposure variable using the respective cut-off values of the subscales of the CTQ. To illustrate the joint distribution of genetic risk (CATT haplotype), childhood maltreatment exposure, and identity dissociation, we constructed a Sankey plot. Additionally, we calculated Odds Ratios (OR) and Risk Ratios (RR) for identity dissociation across combinations of maltreatment exposure and CATT haplotype counts to quantify relative risk patterns. These analyses allow for an examination of the additive and interactive contributions of childhood maltreatment and *FKBP5* haplotypes to the risk of clinically significant identity dissociation within a gene-environment framework.

To explore potential epigenetic mechanisms, we conducted a comprehensive, high-resolution analysis of all 53 available DNA methylation sites across the *FKBP5* locus. Following M-value transformation, we employed Principal Component Analysis (PCA) to extract the primary axes of variance (PC1–PC5) and conducted site-specific linear regressions to test for gene-environment interaction effects (see Supplement for details and null results).

## Results

**Fig. 1:**
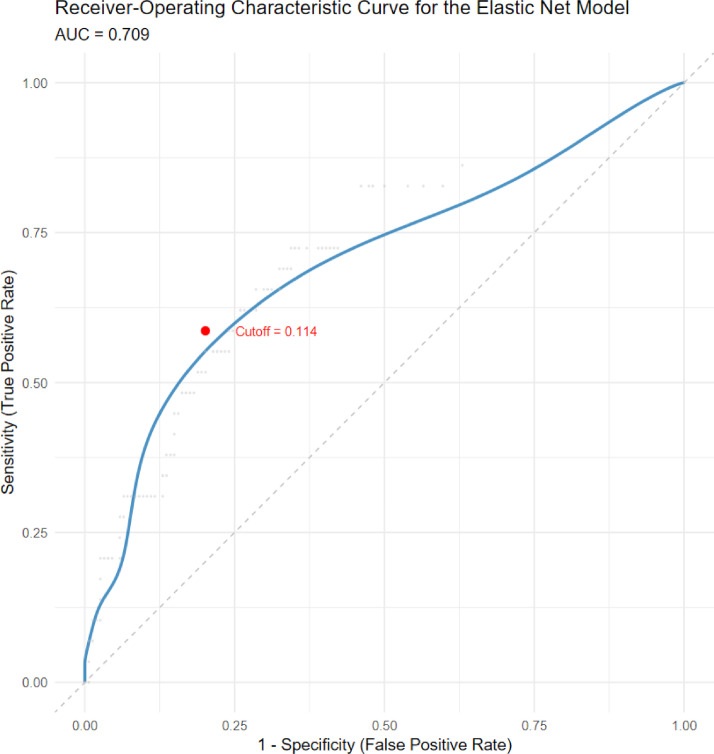
Smoothed receiver operating characteristic (ROC) curve for the elastic net prediction model. The model achieved an area under the curve (AUC) of 0.709, indicating fair discriminatory performance in distinguishing individuals with clinically significant identity dissociation from those without. The curve reflects the trade-off between sensitivity (true positive rate) and 1-specificity (false positive rate) across all possible classification thresholds.

29 out of 183 participants (15.9%) in the validation sample showed meaningful identity dissociation with the criterion of *T* > 70. Means, standard deviations, and correlations with confidence intervals of the variables investigated can be obtained from Table 2. This table also provides a continuous analysis which further validates our model’s structure, as the primary predictors showed consistent linear associations with identity dissociation severity across the entire sample spectrum (*r*s ranging from 0.16 to 0.30 for maltreatment subtypes and *r* = .11 for the CATT haplotype. There were no significant differences in most of the demographic and clinical variables between the training and validation samples (*p* > .05), with the exception of childhood physical abuse (*p* = .01), childhood emotional abuse (*p* = .04), and education level (*p* = .02). For details regarding these differences please see the supplement (supplement Table 1). The Receiver Operating Characteristic Curve for the best elastic net prediction model based on the training sample with an α of 0, reflecting a Ridge regression model including and penalizing all predictors, showed an area under the curve (AUC) for the validation sample of 70.9%. For the optimal cut-off of 0.11, sensitivity was found to be 58.6%, and specificity was found to be 79.9% (see Fig. 1).

**Fig. 2:**
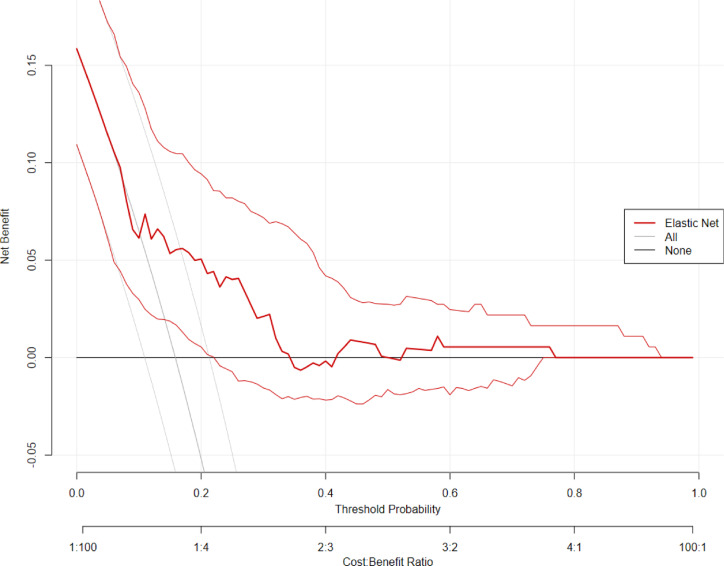
Decision curve analysis (DCA) for the elastic net prediction model. The curve (thick line, slim lines reflecting the 95% confidence interval) illustrates the net benefit of using the model to identify individuals with clinically significant identity dissociation (*T* > 70) across a range of threshold probabilities. The model outperforms simplistic strategies of identifying everyone or noone in a range of thresholds between 6 and 76%, indicating a benefit of the model. This suggests that the model can support decision-making in a wide range of cases from low to quite high probabilities of identity dissociation.

**Fig. 3:**
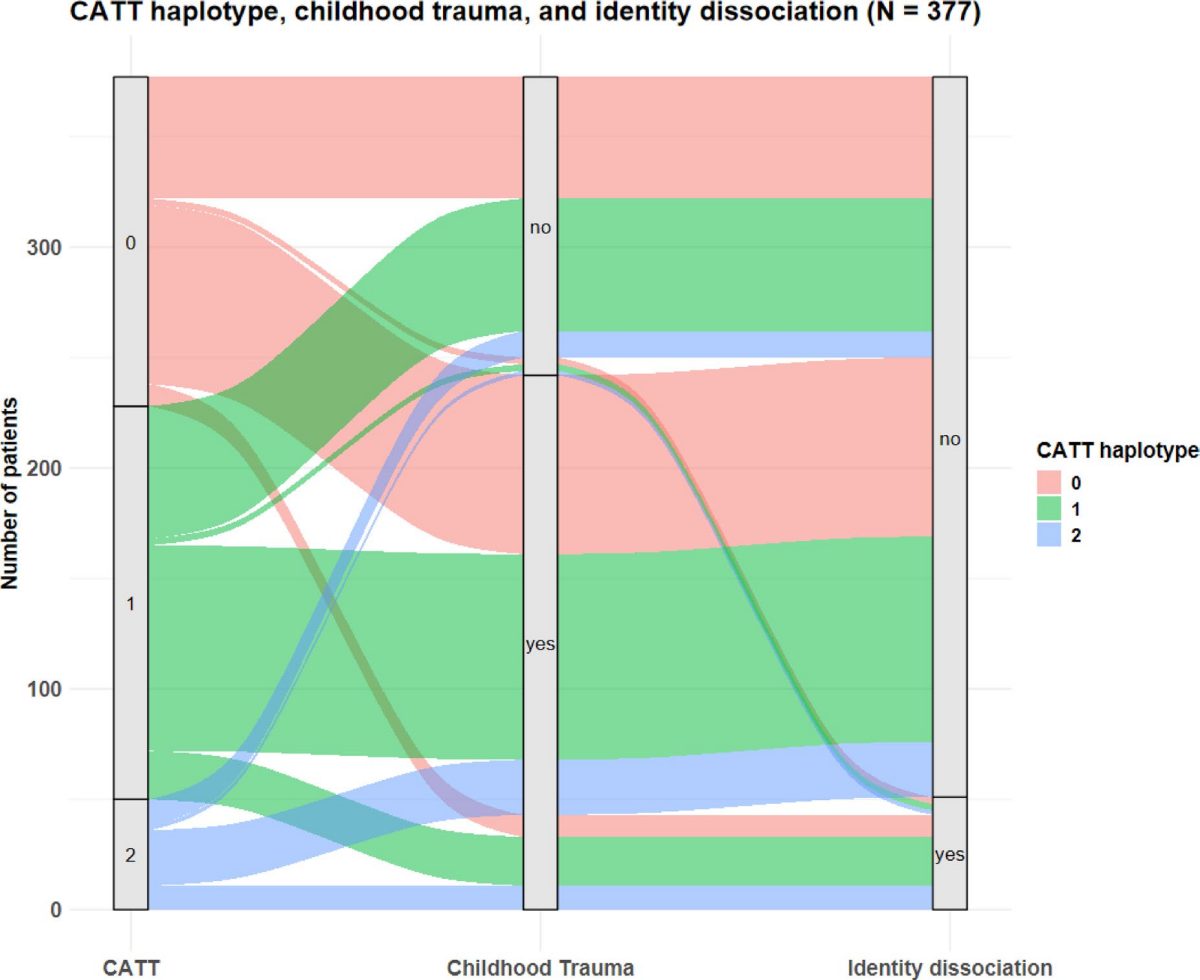
The Sankey Plot groups patients by CATT haplotype count (0, 1, 2), childhood maltreatment exposure (no, yes), and the presence of identity dissociation (no, yes; defined as T > 70 for identity dissociation). The flows represent the number of participants within each transition across categories, with the colors salmon, green, and blue indicating CATT haplotype counts to facilitate visual tracking. This Sankey plot visually highlights how genetic risk and childhood maltreatment exposure jointly relate to the presence of identity dissociation within the sample.

The confusion matrix (please see supplement) showed that the model correctly identified 17 cases of identity dissociation (true positives), mis-identified 31 participants to be cases of identity dissociation when they were not (false positives), missed twelve cases of identity dissociation (false negatives), and correctly identified 123 participants to be no cases of identity dissociation (true negatives). Accuracy of the model was found to be 76.5% (95% CI 69.7–82.4%) and No Information Rate was 0.84. Therefore, the model was not better than a null model always predicting no identity dissociation (*p* = .99). A κ of 0.30 was indicative of a model with fair agreement beyond chance, and MCC was found to be 0.32. McNemar’s test showed that the model was more likely to generate false positives than false negatives (*p* < .001). The positive predictive value was 0.35, and the negative predictive value was 0.91. Detection rate was 0.09. The decision curve analysis (see Fig. 2) showed that the elastic net prediction model yielded a positive net benefit and outperformance over two simplistic default strategies (“treat-all“ or “treat-none“) between thresholds of .06 to .76. The Sankey plot (see Fig. 3) visually highlights how genetic risk and childhood maltreatment exposure jointly relate to the presence of identity dissociation. Table 3 presents the odds ratios and relative risks for identity dissociation depending on CATT haplotype and childhood maltreatment.

**Table 2:** Means, standard deviations, and correlations with confidence intervals.

Variable	*M*	*SD*	1	2	3	4	5	6
1. CTQ emotional abuse	9.20	5.04						
2. CTQ sexual abuse	8.33	5.41	0.59**					
			[0.52, 0.66]					
3. CTQ physical abuse	7.97	3.94	0.71**	0.49**				
			[0.65, 0.76]	[0.41, 0.56]				
4. CTQ physical neglect	6.68	3.10	0.60**	0.45**	0.56**			
			[0.54, 0.66]	[0.36, 0.52]	[0.49, 0.63]			
5. CTQ emotional neglect	9.49	5.03	0.72**	0.50**	0.58**	0.65**		
			[0.67, 0.76]	[0.43, 0.58]	[0.51, 0.65]	[0.59, 0.71]		
6. MDI identity dissociation	1.23	0.58	0.30**	0.25**	0.16**	0.22**	0.23**	
			[0.21, 0.39]	[0.16, 0.35]	[0.06, 0.25]	[0.12, 0.32]	[0.13, 0.33]	
7. Number of CATT haplotypes	–	–	0.12*	0.09	0.11*	0.00	0.06	0.11*
			[0.02, 0.22]	[− 0.01, 0.19]	[0.01, 0.21]	[− 0.10, 0.10]	[− 0.04, 0.16]	[0.01, 0.21]

*M* and *SD* are used to represent mean and standard deviation, respectively. Values in square brackets indicate the 95% confidence interval for each correlation. * indicates *p* < 0.05. ** indicates *p* < 0.01

**Table 3:** Odds ratios and relative risk for identity dissociation depending on CATT haplotype and childhood maltreatment.

CATT haplotypes *N*	Childhood maltreatment	Identity dissociation *n*/*N* (%)	OR (95% CI)	RR (95% CI)
0 (reference)	No (reference)	3/58 (5.2)	Reference	Reference
0	Yes	10/91 (11.0)	2.26 (0.60, 8.60)	2.12 (0.61, 7.40)
1	No	3/63 (4.8)	0.92 (0.18, 4.73)	0.92 (0.19, 4.38)
1	Yes	22/115 (19.1)	4.34 (1.24, 15.16)	3.70 (1.15, 11.85)
2	No	2/14 (14.3)	3.06 (0.46, 20.33)	2.76 (0.51, 14.99)
2	Yes	11/36 (30.6)	8.07 (2.07, 31.47)	5.91 (1.77, 19.75)

To examine the relative contribution of individual predictors, we analyzed the internal structure of the final model (see Table 2 in the Supplement). After standardization of the coefficients to account for the different scales of the predictors, the model revealed that the variables were not equally weighted. The three strongest predictors for identity dissociation based on standardized coefficients were the interaction between Emotional Abuse and CATT haplotype count (β = 0.0012), the main effect of Emotional Abuse (β = 0.0012), and the interaction between Emotional Neglect and CATT haplotype count (β = 0.0011).

Due to the high proportion of female participants (88.6%), the sample was underpowered for a full sex-stratified predictive analysis. However, sex was included as a covariate in the model, showing a negligible relative weight (β = 0.0004) compared to the maltreatment, genetic, and interaction terms (see Table 2 in the supplement).

## Discussion

This study provides novel evidence that clinically significant identity dissociation, a severe form of developmental self-disorder intimately linked to childhood trauma, can be partially predicted through gene-environment interactions involving *FKBP5* haplotypes and adverse childhood experiences. By employing a machine learning approach, we demonstrate the feasibility and boundaries of integrating genetic and environmental markers to identify individuals at elevated risk for identity dissociation within clinical and research settings.

Our elastic net model, which incorporated biological sex, the number of CATT haplotypes within *FKBP5*, childhood abuse and neglect, and their interactions, achieved fair discrimination with an AUC of 70.9%, sensitivity of 58.6%, and specificity of 79.9%. These results indicate acceptable psychometric properties, providing a reasonable capacity to differentiate individuals with clinically significant identity dissociation. However, the model’s relatively low positive predictive value (35.0%) and lack of improvement over the no-information rate underscore a tendency to overpredict, likely reflecting the low prevalence of identity dissociation (15.9%) and inherent class imbalance of the sample. This is further evidenced by the misclassification bias detected by McNemar’s test, where false positives were more frequent than false negatives. Despite these limitations, decision curve analysis suggests that the model offers meaningful net clinical benefit across a wide range of risk thresholds (6–76%), supporting the possible utility in guiding risk evaluation and informing clinical decision-making. Particularly, the model could help identify individuals warranting further focused dissociative symptom assessment, while caution is warranted below and above these thresholds. Notably, the model achieved a high negative predictive value (NPV = 0.91), suggesting its potential utility as a screening tool to rule out individuals at low risk and thus help prioritize limited diagnostic resources. At the same time, the modest classification performance emphasizes the multifactorial and complex nature of identity dissociation. As a complex self-disorder emerging from developmental, neurobiological, cognitive, and social interactions, identity dissociation cannot be fully accounted for by childhood trauma and genetic variation in stress regulation alone. Our findings echo recent systematic reviews that report the absence of consistent genetic biomarkers for dissociation^22,23^ and reflect the broader challenge of predicting dissociative disorders using limited biological and environmental parameters. Clinically, our results highlight the promise of incorporating gene-environment frameworks as adjunctive tools for early risk stratification - particularly in trauma-focused care - rather than as standalone diagnostic instruments. Although our study did not take into account important social and psychological factors such as current stress exposure, medication use, substance use, and sleep problems that may have influenced the development of dissociative phenomena^24,25^, it advances biopsychosocial models of severe dissociation by empirically supporting the interaction between genetic variation in stress response systems and traumatic childhood environments.

Our findings reveal specific gene-environment association patterns. The prominence of childhood emotional abuse and its interaction with the *FKBP5* CATT haplotype as lead predictors suggests a specific neurobiological synergy. From a mechanistic perspective, particularly emotional abuse may act as a potent environmental stressor that, when combined with *FKBP5*-mediated genetic vulnerability, leads to persistent HPA-axis dysregulation and disruptions in neural circuits responsible for memory integration and self-referential processing. While our model utilizes DNA sequence polymorphisms as stable predictors, these genetic variants have been shown previously^26^ to reflect an underlying epigenetic vulnerability. The *FKBP5* CATT haplotype is known to facilitate stress-induced demethylation in intronic regions, a mechanism that leads to the long-term disinhibition of *FKBP5* expression and subsequent HPA-axis resistance. Even though our analysis did not yield positive evidence for this (see Supplement for details and null results), in principle integrating epigenetic variability into future predictive models could significantly refine the biological interpretability and may improve the detection of individuals at risk for severe dissociation.

Several limitations warrant consideration. Most importantly, while we utilized a strict internal split for model training and validation, the lack of an independent external validation cohort limits our ability to confirm the model’s generalizability across different clinical populations. The moderate sample size, although comparable to prior biomarker studies in dissociation, restricts the generalizability of our findings and increases the risk of overfitting despite rigorous cross-validation. Operationalizing identity dissociation via symptom thresholds, while clinically relevant, may insufficiently capture the phenomenological complexity of identity fragmentation across contexts. More conceptually and psychometrically sound assessments of identity fragmentation are needed. Moreover, the absence of multimodal data—such as neuroimaging^27^, physiological markers^28^, or ecological momentary assessments^29^—limits our ability to build more comprehensive predictive models. Similarly, the study did not take into account psychological or other social factors that could have influenced the development of severe dissociative phenomena, such as attachment style, maladaptive cognitions, other or current adverse events, socioeconomic stressors, or clinical factors such as medication use, substance use, or sleep disturbances^24,25,30^.

The convenience sampling and clinical heterogeneity further challenge replicability and external validity. Furthermore, with 88.6% female participants, the study was underpowered to detect sex-specific effects or to build stable independent prediction models for males. Therefore, the generalizability of the current gene-environment model to male trauma survivors remains to be established in more gender-balanced cohorts. Additionally, the lack of structured clinical diagnoses for comorbid conditions, such as PTSD or borderline personality disorder, prevents us from determining the specificity of our model for “pure” dissociation. Instead, our findings likely reflect a transdiagnostic vulnerability across the broader trauma-related spectrum, where identity dissociation serves as a marker for severe psychological disintegration rather than an isolated pathology.

Furthermore, the current study treats the *FKBP5* CATT haplotype as a statistical proxy rather than a direct functional measure. The biological rationale would be further strengthened by integrating functional genomic data, such as per-allele effects on mRNA expression from public eQTL resources (e.g., GTEx, PsychENCODE), developmental expression trajectories (e.g., BrainSpan), or DNA methylation data from larger and heterogeneous cohorts. Future research should incorporate these epigenetic and developmental markers to move from statistical risk prediction to a more granular, mechanistic understanding of how genetic variation and trauma-induced stress-regulation shape the dissociative self-disorder.

Moreover, future research should also replicate and extend our findings in larger, well-characterized cohorts and incorporate multimodal biomarkers and longitudinal designs to unravel the temporal dynamics of gene-environment interplay in identity dissociation. Integrating neural, cognitive, and ecological data with genetic and trauma-related variables promises to refine mechanistic models, advancing early identification, personalized risk stratification, and targeted interventions. Additionally, exploring the adaptive significance and evolutionary “costs” of *FKBP5* haplotypes in diverse environmental contexts could deepen understanding of dissociation’s complex biology and developmental pathways.

In conclusion, despite inherent limitations, this study highlights the potential of gene-environment interactions in predicting identity dissociation and contributes foundational evidence toward unraveling the biological underpinnings of severe dissociative phenomena. Our findings support the integration of genetic, developmental, and clinical perspectives, laying groundwork for future personalized approaches to assessing and treating complex self-disorders linked to trauma.

## Supplementary Information

Below is the link to the electronic supplementary material.

Supplementary Material 1

## Data Availability

The datasets used and/or analysed during the current study are available from Abigail Powers Lott (Co-Director, Grady Trauma Project) on reasonable request.
