# Supplementary material for: Predicting identity dissociation using childhood maltreatment and genetic variation in the stress-response gene FKBP5: a machine learning analysis
Source: Sci Rep. 2026 Mar 6;16:8485. doi: 10.1038/s41598-026-42512-0 (PMC12972126; doi:10.1038/s41598-026-42512-0)
Supplement: Supplementary file 1 — Supplementary Material 1 [file 41598_2026_42512_MOESM1_ESM.docx]

**Supplement Table 1** *Clinical and demographic differences between the training and validation samples*

| **Variable** | **Training Sample** | **Validation Sample** | **Test** | **Test Statistic** | **df** | ***p*** |
| --- | --- | --- | --- | --- | --- | --- |
| Identity Dissociation | M = 1.20 (SD = 0.52) | M = 1.27 (SD = 0.63) | Wilcoxon rank-sum | W = 16,424 |  | .0905 |
| Age | M = 42.01 (SD = 11.92) | M = 39.80 (SD = 11.56) | Wilcoxon rank-sum | W = 19,588 |  | .0823 |
| Income | M = 2.42 (SD = 1.26) | M = 2.20 (SD = 1.38) | Wilcoxon rank-sum | W = 18,718.5 |  | .1258 |
| Childhood Sexual Abuse | M = 8.50 (SD = 5.48) | M = 8.15 (SD = 5.35) | Wilcoxon rank-sum | W = 18,499 |  | .4222 |
| Childhood Physical Abuse | M = 8.41 (SD = 4.23) | M = 7.50 (SD = 3.57) | Wilcoxon rank-sum | W = 20,567.5 |  | .0062** |
| Childhood Emotional Neglect | M = 9.62 (SD = 5.12) | M = 9.36 (SD = 4.94) | Wilcoxon rank-sum | W = 18,140.5 |  | .7062 |
| Childhood Physical Neglect | M = 6.63 (SD = 3.06) | M = 6.74 (SD = 3.15) | Wilcoxon rank-sum | W = 17,202.5 |  | .5436 |
| Childhood Emotional Abuse | M = 9.66 (SD = 5.15) | M = 8.70 (SD = 4.88) | Wilcoxon rank-sum | W = 19,910.5 |  | .0355** |
| Sex | Male = 23 (11.9%) Female = 171 (88.1%) | Male = 20 (10.9%) Female = 163 (89.1%) | χ² | χ²(1) = 0.01 | 1 | .9038 |
| Relationship status | Single, never married = 109 (56.2%) Married = 18 (9.3%) Divorced = 41 (21.1%) Separated = 10 (5.2%) Widowed = 5 (2.6%) Domestic Partner = 11 (5.7%) | Single, never married = 107 (58.5%) Married = 19 (10.4%) Divorced = 39 (21.3%) Separated = 9 (4.9%) Widowed = 2 (1.1%) Domestic Partner = 7 (3.8%) | χ² | χ²(5) = 2.00 | 5 | .8487 |
| Education | **Less than twelfth grade** = 39 (20.1%) 12th Grade/High School Graduate = 55 (28.4%) General Educational Development = 4 (2.1%) Some college or technical school = 49 (25.3%) Technical school graduate = 23 (11.9%) College graduate = 19 (9.8%) Graduate school = 5 (2.6%) | **Less than twelfth grade** = 41 (22.4%) 12th Grade/High School Graduate = 56 (30.6%) General Educational Development = 12 (6.6%) Some college or technical school = 46 (25.1%) Technical school graduate = 5 (2.7%) College graduate = 19 (10.4%) Graduate school = 4 (2.2%) | χ² | χ²(6) = 15.53** | 6 | .0165** |
| Employment | No = 133 (68.6%) Yes = 61 (31.4%) | No = 131 (72.0%) Yes = 51 (28.0%) | χ² | χ²(1) = 0.37 | 1 | .5404 |
| Disability | No = 150 (77.7%) Yes = 43 (22.3%) | No = 155 (84.7%) Yes = 28 (15.3%) | χ² | χ²(1) = 2.55 | 1 | .1104 |
| Identity dissociation | No = 172 (88.7%) Yes = 22 (11.3%) | No = 154 (84.2%) Yes = 29 (15.8%) | χ² | χ²(1) = 1.27 | 1 | .2593 |

**Supplement Confusion Matrix and Statistics**

Reference

Prediction 0 1

0 123 12

1 31 17

Accuracy : 0.765

95% CI : (0.6968, 0.8244)

No Information Rate : 0.8415

P-Value [Acc > NIR] : 0.997389

Kappa : 0.3041

Mcnemar's Test P-Value : 0.006052

Sensitivity : 0.5862

Specificity : 0.7987

Pos Pred Value : 0.3542

Neg Pred Value : 0.9111

Prevalence : 0.1585

Detection Rate : 0.0929

Detection Prevalence : 0.2623

Balanced Accuracy : 0.6925

| **Supplement Table 2** Elastic Net Regression Results for Identity Dissociation | | | | |
| --- | --- | --- | --- | --- |
| Predictor | B | OR | β | OR_std_ |
| Emotional Neglect | 0.0002 | 1.0002 | 0.0010 | 1.0010 |
| Physical Neglect | 0.0002 | 1.0002 | 0.0005 | 1.0005 |
| Sexual Abuse | 0.0001 | 1.0001 | 0.0007 | 1.0007 |
| Physical Abuse | 0.0002 | 1.0002 | 0.0007 | 1.0007 |
| Emotional Abuse | 0.0002 | 1.0002 | 0.0012 | 1.0012 |
| CATT | 0.0012 | 1.0012 | 0.0008 | 1.0008 |
| Emotional Neglect x CATT | 0.0001 | 1.0001 | 0.0011 | 1.0011 |
| Physical Neglect x CATT | 0.0002 | 1.0002 | 0.0009 | 1.0009 |
| Sexual Abuse x CATT | 0.0001 | 1.0001 | 0.0010 | 1.0010 |
| Physical Abuse x CATT | 0.0001 | 1.0001 | 0.0009 | 1.0009 |
| Emotional Abuse x CATT | 0.0001 | 1.0001 | 0.0012 | 1.0012 |
| Male sex | -0.0012 | 0.9988 | -0.0004 | 0.9996 |
| Female sex | 0.0012 | 1.0012 | 0.0004 | 1.0004 |
| Note. B = unstandardized coefficient; OR = odds ratio; β = standardized coefficient (based on 1 SD); OR_std_ = odds ratio per standard deviation. | | | | |

**DNA Methylation**

**Supplementary Methods**

                To ensure a comprehensive assessment of the *FKBP5* locus and to avoid potential bias from pre-defined regions of interest, we analysed all 53 available CpG sites covering the gene. This agnostic, exploratory approach was chosen to detect any localized or regulatory signals that might occur within or outside traditionally studied introns like intron 7 or intron 2 [26]. Methylation beta-values were transformed into M-values $\left( M=\log_{2} \left( \beta/\left( 1-\beta\right) \right) \right)$ to ensure a distribution more suitable for linear modelling. To capture the variance within the locus while accounting for technical noise (typically represented by the first principal component [31]), we performed a Principal Component Analysis (PCA) on the full set of 53 CpGs. The first five principal components (PC1, …, PC5) were extracted for further testing. We tested for a moderated mediation effect using the lavaan package, examining whether the interaction between childhood trauma (CTQ) and the genetic risk proxy (CATT) influenced identity dissociation via the extracted PCs. Furthermore, a high-resolution site-by-site scan was performed, where each of the 53 CpG sites was individually tested for a $CTQ x CATT$interaction effect using linear regression.

**Supplementary Results**

                The agnostic, exploratotry analysis of the 53 *FKBP5* CpG sites did not reveal any significant epigenetic associations. The primary direct effect of childhood trauma on identity dissociation was robust $\left( \beta_{\mathrm{std}} = 0.235, p < .001 \right)$, but no significant indirect effect through any of the five principal components was observed (all paths p > .25). This confirms that even when considering the total variance of the locus, no mediation of the interaction occurs. In the site-by-site analysis, no CpG site reached significance after Bonferroni correction for 53 tests (all$p_{adj}=1.0$). Only two sites showed nominal significance ($p < .05$, but uncorrected), which is consistent with the expected FPR under the null hypothesis.

**Supplementary Discussion**

The lack of significant findings is likely attributable to the restricted inter-individual variability in DNA methylation within this cohort (mean variance across all 53 sites: 0.00178). This high degree of epigenetic homogeneity at the *FKBP5* locus limited the statistical power to detect subtle regulatory changes, suggesting that in this specific sample, the clinical effects of childhood trauma on dissociation are not mediated by *FKBP5* methylation.
